# The Comparative Psychology of Intelligence: Some Thirty Years Later

**DOI:** 10.3389/fpsyg.2020.00973

**Published:** 2020-05-19

**Authors:** Irene M. Pepperberg

**Affiliations:** Department of Psychology, Harvard University, Cambridge, MA, United States

**Keywords:** comparative psychology, avian cognition, animal intelligence, grey parrot, animal cognition

## Abstract

After re-reading Macphail’s (1987) essay “The Comparative Psychology of Intelligence” with all the associated commentaries, I was struck by how contemporary many of the arguments and counter-arguments still appear. Of course, we now know much more about the abilities of many more species (including their neurobiology) and fewer researchers currently favor explanations of behavior based solely on associative processes; however, the role of contextual variables in comparative psychology still remains cloudy. I discuss these issues briefly. Given my research interests involving the cognitive and communicative abilities of Grey parrots, the one aspect of the original article upon which I feel I can comment in depth involves Macphail’s claims about the importance of language—and specifically syntax—in problem-solving and thus in placing humans above all other creatures. Granted, no other species has (or in my opinion is likely ever to acquire) everything that goes into what is considered “human language.” Nevertheless, several other species have acquired symbolic representation, and considerable information now exists upon which to base an argument that such acquisition by itself enables more complex and “human-like” cognitive processes. Such processes may form the basis of the kind of intelligence that is measured—not surprisingly—with human-based tasks, including the use of such representations as a means to directly query non-human subjects in ways not unlike those used with young children.

## Introduction

Over 30 years have passed since Macphail’s essay “The Comparative Psychology of Intelligence” was published in *Behavioral and Brain Sciences* along with numerous commentaries, critiques, and his rebuttals (Macphail, 1987). With some exceptions that I will not discuss below (e.g., the most notable being that recent decades have seen an unprecedented upsurge in both the reputation and number of publications in comparative psychology, in contrast to the adverse trends upon which Macphail commented in 1987—e.g., the emergence of a new journal, *Animal Cognition*; the independence of the *Journal of Comparative Psychology*; the founding of the *Comparative Cognition Society;* the publication of Call et al.’s 2017 *APA Handbook of Comparative Psychology*), many of the same arguments and counter-arguments might be found in contemporary literature. Granted, we now know much more about the neurobiology of many more species (particularly the fairly recent findings about the complexity of psittacine and corvid brains) and have reams of data about sophisticated abilities of previously unstudied or rarely studied creatures such as reptiles and even invertebrates. Too, a larger number of researchers are now less likely to reduce the characteristics of the tasks being studied to chains of associative processes, though some still disagree. The extent to which the effects of specific capacities that have been shaped by evolutionary pressures can be defined or explained by contextual variables remains cloudy; for example, some researchers propose that many living creatures begin life with certain equivalent core abilities, upon which more complex cognitive capacities may be built to varying degrees. I’ll briefly discuss a few of these topics, then concentrate on what for me is a central issue—that of the effects of the acquisition of symbolic communication, albeit something much less than language, on the cognitive capacities of non-human subjects, and how such communication may expedite the study of such capacities. Almost all my comments will arise from the standpoint of a researcher on such avian capacities.

## Is Everything Reducible to Associations?

This basic claim of Macphail is controversial, specifically because the answer to the question depends solely on one’s theoretical framework. Some researchers still argue that all complex tasks can be reduced to a series of associations and others heatedly disagree—see, for example, Heyes (2016) associative-learning based arguments for the explanation of human imitation, other researchers’ counter-claims for neonatal imitation (Meltzoff and Moore, 1999; Simpson et al., 2014), and the demands of still others for further research (Vincini et al., 2017). Despite an overall lack of consensus on exactly what does separate associative versus other forms of learning, many researchers seem to agree on mental representation, rule-based learning and symbolic processing as behaviors that differ in measurable ways from associative learning (e.g., McLaren et al., 2018; Church, 2019; Smith, 2019; Wills et al., 2019). Note that Macphail acknowledges only the uniqueness of language, of which symbolic processing is merely one aspect. The debate framed by Macphail thus clearly has not been resolved, but its parameters have widened considerably, with many cogent arguments for multiple levels of processing. I leave it to colleagues who specialize in areas outside of symbolic processing to address those aspects of the debate in full.

## What Is the Role of Neuroanatomy?

As with the debate on associative learning, my knowledge of neuroanatomy is limited compared to that of others who will also likely be commenting on Macphail’s paper. However, I wish to note, if only briefly, that the explosion of information on what is now known about non-human brains cannot be ignored, particularly with respect to avian cognition. Numerous papers have demonstrated that the architecture of neither a primate nor even a mammalian brain is required for complex cognitive processing (e.g., Iwaniuk et al., 2005; Jarvis et al., 2005; Güntürkün and Bugnyar, 2016; Gutiérrez-Ibáñez et al., 2018; Rinnert et al., 2019). Significantly, Olkowicz et al. (2016) found that parrots and corvids in particular have forebrain neuron counts equal to or greater than primates with much larger brains, and suggest that such avian neural densities are likely responsible for their high levels of intelligence. A recent study even argues for adult neurogenesis in the Grey parrot brain as being correlated with advanced cognitive processing (Mazengenya et al., 2018). Other studies demonstrate certain relationships (and thus suggest the possibility of shared forms of processing) among avian, mammalian, and reptilian brains based on common ancestry (Tosches et al., 2018). Additional research (see entries in the aforementioned *Handbook*) demonstrates many non-human abilities that compare favorably with those of humans. However, lest we are tempted to use this information to argue for the lack of differences between human-non-human abilities or among non-human species, we also know that even within related species—for example, among closely-related corvids and also among less-closely related parrots—there exist subtle and not-so-subtle brain variations, respectively, that likely are correlated with differences in the types and extent of processing abilities (see Basil et al., 1996; Gould et al., 2013; Chakraborty et al., 2015). Differences in the relative sizes of specific neural areas, the presence or absence of specific areas, and the overall internal organization will affect the complexity of a species’ cognition and memory. Notably, even within the same species, individual differences exist with respect to competencies: we argue about human brilliance based on outliers (e.g., Einstein, Beethoven, Rembrandt, Shakespeare), but the ‘average’ human clearly does not exhibit such capacities, even when contextual variables are taken into account. Although we have not entirely determined the neural correlates of human intelligence (see Rhein et al., 2014; Wen et al., 2019), evidence for inter-individual differences obviously exists, both in brains and behavior. Surely interspecies differences can be at least as great as intraspecies ones?

## Species Differences in General?

At least one reptile fails a task in simple numerical cognition (although not in distinguishing larger from smaller objects; Petrazzini et al., 2017), and comparative work by researchers (e.g., Kamil and his students; see Olson, 1991; Olson et al., 1995) have shown that some bird species excel at certain spatial learning tasks and not on others such as match-to-sample. Some of these behavioral differences may be related to differences in brain structure (see previous section), but one might, like Macphail, argue that such differences are simply a matter of “contextual variables.” Interestingly, some researchers now argue, consistent with Macphail’s claims, that most species have very similar, basic “core” capacities, which are involved in representing certain aspects of objects, actions, number, space, and (possibly to a lesser extent), social interactions (Spelke and Kinzler, 2007)—that is, diverse species show remarkably similar levels of competence on a number of rather basic tasks. However, these so-called *core* capacities, which are present in most species at a very young age, are but the building blocks of complex cognitive processing. If all that are being studied are tasks that rely on these core capacities (e.g., more-less, object identity), then the few differences that emerge are, indeed, likely to depend on contextual variables. However, contra Macphail, additional research demonstrates that different species develop additional abilities, beyond those based on these core capacities, to different extents: if the tasks being studied require more than core capacities, differences exist in various abilities to process more and more complex information (Wright et al., 2018). Specifically, complex cognition is not only the ability to come to a decision by evaluating, or processing, current information on the basis of some representation of prior experiences (e.g., Kamil, 1984). Complex cognition must also include the capacity to choose, from among various possible sets of rules that have been acquired or have been taught, the set that appropriately governs the current processing of this data—that is, in order to solve a problem, the subject must first decide which rules are appropriate for the processing of data (i.e., determine which of many possible different types of problem is being posed) and then figure out what types of data are needed for the solution. According to this criterion, a subject that is limited to organizing information on the basis of a single set of rules—a subject that has little more than the core capacities that allow success at something like a matching procedure—will not have the occasion to demonstrate complex cognitive processing (Pepperberg, 1990).

*How* a subject develops more advanced capacities from core capacities, and exactly *why* different species acquire more advanced capacities than others in some domains and not in other domains—these questions still require complete answers. The answers likely lie in some confluence of evolutionary pressures in the form of environmental input and the wherewithal to process the information in this input, and thus in some differential aspect of neurobiology—but remain a subject for study at present.

## The Role of Language

Macphail argued that although no differences exist in the intelligence of the various non-human species, he made a special case for humans based on their acquisition of language, particularly syntax. The extent to which Macphail’s arguments are still valid enter yet another murky realm. He argued that, in almost all cases, what passed for “language” in the various non-human programs he reviewed (e.g., Gardner and Gardner, 1969; Premack, 1971; Rumbaugh, 1977; Patterson, 1978; Terrace et al., 1979; Savage-Rumbaugh et al., 1983) were simple associations between objects and artificial symbols; he argued that these subjects’ inability to create novel sentences meant that whatever success they had achieved was insufficient to raise their intellectual capacity. By ignoring some possible instances of novelty (e.g., Fouts and Rigby, 1977; Rumbaugh, 1977) and—most importantly—only briefly noting work on cetaceans (e.g., studies by Herman and Schusterman and their students), Macphail gave insufficient credit to these species’ abilities to understand certain levels of rule-governed behavior. Herman’s dolphins, for example, could respond with statistically significant accuracy to *novel* 5-element sentences such as “modifier + direct object + verb + modifier + indirect object” (e.g., fetch the right hoop—as opposed to the left one—and bring it to the top frisbee—as opposed to the bottom one; or could swim through both hoops in the given order; Herman et al., 1984; see Schusterman and Gisiner, 1988, for related work on sea lions).

To give Macphail credit, rule-governed behavior is only a simple form of syntax, and no non-human has demonstrated capacities fully comparable to all the possible intricacies of complex human communication. It must be noted, however, that not every human language includes all those intricacies—e.g., some lack complex embedding and constructs such as the passive (e.g., Everett, 2005; some controversy surrounds that claim, but Huttenlocher et al., 2010 has shown that when raised in impoverished settings, even American children’s sentence structure lacks such complexities). Clearly, cetaceans in such training programs demonstrated far more complex behavior patterns than would be possible without such instruction. Notably, reducing their behavior to chains of association would require the same type of reduction for much of human language, a communication system that Macphail argued was unique.

Macphail also failed to fully appreciate what I had recently accomplished with a Grey parrot (Pepperberg, 1983), research that also contradicted claims of simple association rather than full referential abilities. The parrot, Alex, by showing that he could—even at that early stage in his training—vocally indicate different attributes of a single item (its color, shape, material, and overall label), demonstrated that, even for a novel item, he could interpret the various possible questions that could be posed (i.e., determine which attribute was being targeted), search his repertoire for the set of labels that were hierarchically organized under that attribute (e.g., if the question was “What color?,” know to examine labels such as yellow, blue, green, etc. instead of paper, cork, wood, etc.), chose the one appropriate label, and then encode it vocally.

Of particular interest is that Premack (1983) had made a somewhat similar argument to that of Macphail concerning the effect of language on cognition but had come to a strikingly different conclusion. Premack (1983), sidestepping the controversy surrounding whether animals were capable of human-level language, claimed that non-humans who learned *symbolic representation*—in which a non-iconic symbol stands for an object, an attribute, an action, etc.—have an enhanced ability to perform tasks that require abstract thinking. He buttressed these claims with data demonstrating that those of his apes that had acquired such symbolic representation outperformed those that did not, particularly on tasks such as analogical reasoning. [Interestingly, although some evidence exists for such reasoning in subjects lacking symbolic representation (e.g., a relational MTS task, see data and discussion in Obozova et al., 2015), those claims have been critiqued by Vonk, 2015]. I discuss several experiments from my laboratory that give additional credence to Premack and suggest the limits of Macphail’s argument. I do so with the following caveat: I have argued previously (summarized in Pepperberg, 1990) that training on symbolic representation (or its lack) is likely to affect only the ease with which animals learn and can be tested on certain concepts. I’ve also argued that although a system of two-way communication may enable a researcher to teach a concept that an animal subject may not easily acquire by other means, such acquisition is unlikely if the animal does not have the basic cognitive capacity for such acquisition. The first set of experiments I review relates to these points. However, I now am not entirely sure about a corollary of those two claims—that acquisition of symbolic representation does not affect how subjects manipulate information—I believe that, in some specific instances, such a change in processing ability may exist. Possibly, once a non-human understands that a symbol can be used to represent an object or an action, it can then understand how, for example, a three-dimensional entity can be represented by a two-dimensional one (e.g., optical illusions, Pepperberg and Nakayama, 2016) or that two symbols (e.g., one vocal and one visual) that separately represent the same object can then represent each other (a formal equivalence, Pepperberg,2006b), or that symbols can be used as place-markers to assist in tasks requiring memory and evaluation of probability (Pepperberg and Pailian, 2017; Clements et al., 2018). I will discuss one such topic—various studies on numerical concepts—in depth. I will also suggest some limits to the functionality of symbols, based on the extent of their use and comprehension (Bowden et al., 2019).

### When Symbolic Representation Affects Training/Testing and Therefore Results

I describe two studies in depth in which the use of symbolic processing plays an important role, not specifically because such representation allows for more complex processing, but because it facilitates training and/or eliminates confounds that can detract from the claims of success in other species. In those as well as the additional studies briefly referenced above, I have demonstrated that Grey parrots have succeeded on certain tasks that have proven challenging to other non-humans; I believe that the success of the birds may rely on their access to symbolic representation. For these and other tasks, exact comparisons between non-humans with and without symbolic representation cannot be made specifically because the parrot can choose one vocal response from its entire repertoire, much like children but unlike almost all other non-humans. These findings do not support Macphail’s claims of a lack of difference in intelligence among species, but rather suggest that having even some language-like elements may be instrumental in assisting researchers to explore these differences.

#### A Study on Abstract Relations: Bigger/Smaller

The ability to predicate a response on a relational rather than an abstract basis is frequently used as a metric for comparing cross-species abilities, because understanding relations (darker than, bigger than, etc.) is supposedly a more complex task than learning to respond to an absolute concept (e.g., redness; see discussions in Schusterman and Krieger, 1986; Pepperberg and Brezinsky, 1991). Responding on a relative basis requires a subject to compare stimulus choices and then derive and use an underlying, more abstract (and thus general) concept; it is the comparison that is crucial, because in a task such as “lighter than” the right answer in one trial (“gray” in a task pitting black against gray) may be the incorrect in the next trial (pitting white against gray). In contrast, learning an absolute stimulus value requires only that a subject form a single association (e.g., choose gray; Thomas, 1980). Because tasks that involve relative concepts often allow organisms to learn something about both absolute and relative concepts concurrently (Premack, 1978), researchers who use such tasks for cross-species evaluations of cognitive capacity must determine the extent to which their subjects rely on relative information in problem-solving. I’ll present a few examples without going into a detailed review of the literature.

It is not that subjects unable to use symbols completely fail in demonstrating relative concepts, but rather that these subjects tend to focus primarily on absolute concepts and that demonstrating their understanding of relational concepts can be challenging. In one set of such studies, starlings that are taught to discriminate a set of rising tones from a set of descending tones and are then asked to transpose to a novel set in a totally different key, can transfer solely under very specific conditions. Such data show that they respond on a relative basis as a secondary strategy, only after acquiring information on an absolute basis (Hulse et al., 1984, 1990; Cynx et al., 1986; Page et al., 1989; MacDougal-Shackleton and Hulse, 1995). This so-called “frequency range constraint” may derive from ethological priorities, where changing the overall pitch changes the meaning and importance of the signal (see discussion in Pepperberg, 1999).

Other studies have examined relative luminance or size. Here, pigeons also tend to discriminate on the basis of absolute, rather than relative, brightness, and rhesus monkeys do the same for both brightness and size, although changes in experimental design (e.g., how stimuli are presented) may result in their showing some understanding of the relative concept in transposition trials (see Pasnak and Kurtz, 1987; Wills and Mackintosh, 1999). Interestingly, horses seem capable of size transpositions, although—like almost all the other subjects tested—only for the direction in which they were trained—that is, to stimuli that are either relatively larger or relatively smaller, but not to both within the same experiment (Hanggi, 2003). And, of course, responding to “larger” is only meaningful if a subject can also respond to “smaller,” so that the task is not simply the ability to respond to “more” (possibly a preference related to foraging).

Two studies with symbol-using subjects, on relative size, however, demonstrate the worth of symbolic representation. Here, the subject is taught a label for both of the concepts that are being tested, rather than having to derive the concepts over large numbers of trials. In one study on a sea lion, Schusterman and Krieger (1986) demonstrated that their subject understood the concepts of both bigger and smaller and transposed to objects of novel sizes, such that the previously correct choice would now be incorrect; their subject, however, was not tested on items completely different in shape or material from those used in training. My Grey parrot, Alex, after learning to respond to “What color bigger/smaller?” for three sets of items, was able to transfer, without additional training, to a large number of sets involving sizes outside the training paradigm and to totally novel objects with respect to shape, color, and material; he also spontaneously transferred to the questions “What matter bigger/smaller?” and, when the two objects were of the same size, spontaneously responded “none,” transferring his understanding of that label from a study on a lack of same/difference (Pepperberg,1987a, 1988; Pepperberg and Brezinsky, 1991). That these subjects had the ability not only to respond to the largest *or* the smallest item that was present, but also could recognize that on *any* trial, *either* bigger or smaller could be queried, demonstrated a far greater understanding of the relative concept than had been shown by any other non-humans. Their training in symbolic representation was not likely responsible for this understanding but enabled them to display such capacities at a higher level.

#### The Müller-Lyer Illusion

Few studies that examine how non-humans perceive optical illusions are directly comparable to those with humans. Grey parrots that have some referential use of English speech, however, allow for such comparative studies, as these birds can be tested just as are humans, by asking them to describe exactly what they have seen. Specifically, no studies had previously been performed on an avian subject that, *without any training on the actual task*, could—as would be possible for Alex—simply state vocally whether or not an optical illusion had been observed. My colleagues and I began a series of such studies by examining the Müller-Lyer illusion (Pepperberg et al., 2008) because it is well-represented in the scientific literature; in the classic form, humans underestimate or overestimate the length of a line that has arrows attached, respectively, either inwardly < > or outwardly > <. Many explanations exist as to why humans are subject to the illusion (see review in Pepperberg et al., 2008), but our main interest was in determining how it would be processed by the avian visual system, which is notably anatomically and neurobiologically distinct from that of humans (see review in Shimizu et al., 2010 for both similarities and differences). Would a parrot, separated from humans by 300 million years of evolution (Hedges et al., 1996) also be duped into thinking that the two horizontal lines in the illusion differed in length because of the placement of the arrows?

Some evidence existed for the illusion in ring doves (Warden and Baar, 1929), pigeons (Nakamura et al., 2006), and chickens (Winslow, 1933), but the data were not conclusive. Intensive training procedures were generally necessary to enable these birds to discriminate the initial stimulus and subjects were then tested on their recognition of similar patterns. Results often depended on, for example, statistical averaging over 100s of trials of pecking/touching behavior to a very limited set of choices and thus were often highly variable and dependent upon details of the experimental design (reviewed Pepperberg et al., 2008). Rosa Salva et al. (2014) clearly discuss how intensive training and the requirement that subjects perform such physically manipulative responses result in failures that may be avoided when subjects engage in incidental learning and can respond in a more naturalistic manner. Thus, having a subject that, like humans, would have an extensive vocal repertoire from which it could choose any utterance (from over 100 possibilities), and could simply be asked to describe what it sees, on only a few trials per type of stimulus, without any prior training on any materials related in any way to those stimuli, would avoid these issues. Also, because such vocal responses and lack of extensive training did enable us to query Alex only a relatively few times compared to other non-humans, we could prevent, at least to some extent, a decrement in perceiving the illusion that can occur over time (Mountjoy, 1958; Predebon, 1998, 2006).

Alex was shown the Brenano version of the task (>–<–>), considered to be equivalent to the presentation of two separate figures (e.g., Sadza and de Weert, l984), to ensure that he focused on both illusions simultaneously; the horizontal lines were of different colors and he was queried as to “What color bigger/smaller?,” a concept he already understood (Pepperberg and Brezinsky, 1991). Controls were lines with the arrows replaced by perpendicular lines; if he saw the illusion as do humans, he would give a color response on the standard queries and say “none” (his response to the absence of a size differential) in the controls. Requiring responses with respect to both bigger and smaller forced Alex to attend to and interpret each question individually, unlike most other non-human subjects. To test the extent to which he saw the illusion, we varied the pitch of the arrow from the standard 45° and the thickness of the horizontal lines. Again, if Alex responded as did humans, thick horizontal lines would decrease the extent to which he saw the illusion, as would angles that approached 90°.

Alex’s data were scored as ‘illusion reported’ if he named the shaft color that human observers would report, as ‘no illusion’ if he reported “none,” and ‘opposite the illusion’ if he reported the color opposite to the illusion response. After accounting for mistrials due to inattention, he reported the illusion in about 88% of the trials in which human observers would have reported the classic illusion (such a rate is consistent with his overall accuracy in color labeling, 80–85%; Pepperberg, 1999) and he showed a lessened or absent illusion in control trials where humans would not have reported the illusion, as the line thickness increased and the arrow angles altered (Pepperberg et al., 2008). Interestingly, even with the relatively limited number of trials we administered, we needed to account for some habituation and decrement of response due to inattention; such findings suggest how the effects of extended training and testing may have affected the results of previous studies.

His data suggest that even if avian systems for visual input differ from those of mammals, similar processing may occur within various neural structures. The importance of the data, however, lie not only in finding out how a Grey parrot perceives the world in which we co-exist, but also in being able to compare his responses *directly* to humans who view the same stimuli. This study emphasizes ways in which symbolic representation affects *how* non-humans can be tested on certain concepts. Thus, although one may argue that this study involves perception more than intelligence, my point is that the results show that symbolic representation enables us to directly compare how perceived information is processed, which is part and parcel of intelligence.

#### Other Studies

Alex was tested on many other types of tasks, including the concept of same-different (Pepperberg,1987a). A review of that entire topic is the basis for a separate paper (Pepperberg, in review), but the central issue is as follows. Same-different is more than identity versus non-identity or the difference in entropy between stimuli (e.g., Young and Wasserman, 2001). Rather, it is a task that, according to Premack’s (1983) stringent criteria, requires a feature analysis of the objects being compared, recognition that objects can simultaneously exhibit attributes that involve *both* similarity and difference, and the ability to understand which attributes are being targeted based on questions of either similarity *or* difference. Because an appropriate response requires that a subject (a) attend to multiple aspects of two different objects; (b) determine, from a verbal question, whether the response is to be based on sameness *or* difference; (c) determine, from the exemplars, *exactly* what is same or different (i.e., what are their colors/shapes/materials?); and then (d) produce, verbally, the label for the hierarchical category of the appropriate attribute, the task is another instance in which symbolic representation is likely critical for success (Premack, 1983).

Furthermore, Alex was not the only bird that my students and I have studied; numerous experiments on another Grey parrot, Griffin—one with a less extensive repertoire but with otherwise similar experiences to those of Alex—provide further evidence for the importance of symbolic representation in the study of non-human intelligence. Detailed descriptions of such studies with respect to the importance of symbolic representation are reviewed elsewhere (Pepperberg, in press) but, as noted above, his demonstration of capacities comparable to that of a 7-year-old child on topics such as Piagetian probability (Clements et al., 2018) likely involved his understanding that symbols can be used as place-markers to assist in tasks requiring memory and evaluation of chance. His ability to label occluded objects correctly and recognize Kanizsa figures also likely depended upon his symbolic understanding, in those instances for transferring his knowledge that a three-dimensional entity can be represented by vocal label into knowledge that the same entity can also be represented by a two-dimensional depiction (Pepperberg and Nakayama, 2016).

### When Symbolic Representation May Enable Advanced Information Processing

For the topics discussed and referenced above, non-humans without symbolic representation were often able to demonstrate certain levels of competence, but non-humans with such representation were able to demonstrate either somewhat higher levels of such competence or were able to demonstrate their competence simply more efficiently. For the topic discussed below, the data suggest that the capacity for symbolic representation may actually have affected whether the non-human subject actually *could* demonstrate the given capacity; non-humans lacking the levels of Alex’s representation have not, at least at present, shown such levels of intelligence.

#### Exact Numbers, Including a ‘Zero-Like’ Concept

Numerical competence can be defined so as to include a wide range of abilities, ranging from a simplistic understanding of more-versus-less to full comprehension of various forms of set theory. What makes numerical competence interesting as an overall topic is that number is not an inherent attribute of an object, as is color, shape, or material, but rather a descriptor that is applicable to any discrete collection of entities. What makes numerical competence relevant to the theme of this paper, however, is its relationship to symbolic representation, and the argument that a full understanding of number begins with the ability to use symbols to designate exact quantities (see Wiese, 2003).

It is, of course, true that some basic understanding of number is a widespread phenomenon; use of a primitive, approximate number system (ANS) has been observed in almost every species examined, from fish (Petrazzini et al., 2015) to bears (Vonk and Beran, 2012), from preverbal children (Wynn, 1990) to preliterate hunter–gatherers (Frank et al., 2008) (but note reptile exception above, Petrazzini et al., 2017). Although the ANS allows for some level of numerical discrimination, ANS tasks are not symbol-based and precision under the ANS decreases sharply in all mathematical operations as the number of items involved increases; for example, accurate comparison of two numerical sets is possible only when they differ by a sufficient ratio (Halberda et al., 2008). Consequently, species possessing an ANS can generally choose the greater of sets consisting of one, two and three items exactly, but their accuracy decreases when larger numbers and smaller ratios are involved and the ANS is not useful when discrimination among sets of even moderately larger quantities (e.g., eight versus nine) is required to solve a problem or to achieve success on a task.

In contrast, symbolic representation of number—the understanding that individual symbols represent exact, specific quantities—enables advanced capacities such as counting principles, precise addition, subtraction, etc. Acquisition of symbolic representation of number is a slow, multi-year process, even for human children (Fuson, 1988; Carey, 2009), and was once thought limited only to humans (reviewed in Pepperberg and Carey, 2012). Notably, Hurford (1987) and Dehaene (1992) have also suggested a close correlation between labeling and number skills, in the sense that numerical cognition is “a layered modular architecture, the preverbal representation of approximate numerical magnitudes supporting the progressive emergence of language-dependent abilities such as verbal counting” (Dehaene, 1992, p. 35).

Indeed, only a very few non-humans have acquired exact symbolic number representation: two apes, Matsuzawa’s Ai (Matsuzawa, 1985) and Boysen’s Sheba (Boysen and Berntson, 1989), and my subject, the Grey parrot Alex (Pepperberg,1987b, 1994). Sheba’s instruction on symbolic representation primarily involved use of physical Arabic numerals; in contrast, at the time that their numerical training began, Ai and Alex had already been trained to identify objects and colors and Alex had also begun to recognize shapes based on their numbers of corners (Asano et al., 1982; Pepperberg, 1983). Thus, these two latter subjects had to reorganize how they categorized objects in their world. They had to learn that a new set of labels, either physical symbols or the vocal labels “one,” “two,” “three,” and so forth represented a novel classification strategy; that is, one based on both physical similarity within a group (e.g., that the objects were, for example, all keys) and a group’s quantity (the exact number of a set), rather than solely by physical characteristics of group members (being metal). They also had to generalize this new class of number labels to sets of novel items and items in random arrays; Alex, unlike other subjects, also had to extend his understanding to heterogeneous collections. All three subjects eventually expanded their competency to more advanced numerical processes (Pepperberg, 1999, 2006a). And, as we shall see, they all understood, at least to some extent, that numbers are flexible tools that can be used to assess both cardinal and ordinal relations (Wiese, 2003). Such behavior is not easily reducible to simple associative learning; however, these competencies were acquired by subjects who had not demonstrated communication skills comparable to human language.

All three of these subjects acquired the ability to identify sets of objects exactly (i.e., their accuracy did not decrease as the size of the set increased as in the case of the ANS). Initial studies showed that Sheba and Alex could quantify sets up to six (Pepperberg,1987b; Boysen, 1993), and that Ai could distinguish sets up to nine, although for the largest quantity she seemed to use a fairly accurate form of estimation rather than counting (Biro and Matsuzawa, 2001a). All three subjects were equally accurate when asked to examine novel sets and sets placed in random arrays. Such behavior is not possible without the use of symbolic representation. Interestingly, when asked to distinguish between numerical sets, data from monkeys without such training obeyed ANS rules (Brannon and Merritt, 2011), whereas those with symbolic representation were considerably more accurate, particularly when the two sets were large and differed by one unit (Livingstone et al., 2010).

The two apes and the parrot also acquired a zero-like concept. Ai and Sheba were specifically trained on the concept (Boysen and Berntson, 1989; Biro and Matsuzawa, 2001b). Alex, in contrast, spontaneously transferred his use of “none,” which he had originally learned to produce so as to designate the absence of a common attribute in a same/different task (Pepperberg, 1988), to now designate the absence of a set of objects (“none” present, Pepperberg and Gordon, 2005). Apropos of the topic of this review, such a transfer was possible *only* because Alex could access his entire verbal repertoire during sessions—that is, was not limited to choosing among a small number of possible response keys—and, in this case, was also able to use his vocal abilities to manipulate the experimenter into asking him the question that led to his demonstrating this transfer (Pepperberg and Gordon, 2005). Again, he had received no training on use of any symbol, vocal or physical, to represent absence of quantity.

Alex, without training, was also able to quantify subsets in a heterogeneous array: given four groups of items that varied in two colors and two object categories (e.g., blue and red keys and trucks), he was able to label the number of items uniquely defined by the conjunction of one color and one object category (e.g., “How many blue key?”) with an accuracy >80% (Pepperberg, 1994). Notably, the study replicated work with adult humans (Trick and Pylyshyn, 1989), who use an exact, rather than an ANS, in this task. Young children who, like Alex, had been taught to label homogeneous sets exclusively, may fail this task; they may be at the stage where they can label exact quantities, but often give the total number of items instead of that of the targeted subset (see Siegel, 1982; Greeno et al., 1984). Interestingly, *unlike the other subjects*, Alex was never trained on number comprehension; nevertheless, when tested, his comprehension accuracy was somewhat superior to that of production (Pepperberg and Gordon, 2005). Again, such abilities are based on symbolic representation.

#### Ordinality

Whereas the apes had been trained from the start of their studies to use Arabic numerals, and learned their quantifications in numerical order, much like children (see Carey, 2009), Alex had been trained only on vocal numerical labels, and, in contrast, first learned “three” and “four” (simultaneously), then “two” and “five” (again, simultaneously), and lastly “six” (pronounced “sih”) and “one” (again, simultaneously). Note that, unlike the other subjects, he couldn’t simply point to an answer. Instead, for each label, he had to learn to configure his vocal tract appropriately (see Patterson and Pepperberg, 1994, 1998), a somewhat difficult process (Pepperberg, 1999). After demonstrating his accuracy—as noted above, *without any training*—on comprehension (Pepperberg and Gordon, 2005), he was then taught to identify Arabic numerals (production and comprehension), using the same labels as he had used for numerical sets of objects, *but in the absence of any of these sets*. Thus, given a tray containing all the plastic or wooden Arabic numerals of different colors from 1 to 6, he learned to correctly respond to queries of, for example, “What color is ‘four’?” or “What number is ‘blue’?” Of particular interest is that he then *spontaneously* inferred the ordinality of his labels, as tested by his stating the color of the larger or smaller Arabic digit in a paired set or “none” if they represented the same quantity (Pepperberg,2006c). These data also demonstrated that he was capable of a formal stimulus equivalence (Sidman et al., 1989), a behavior that is again dependent upon symbolic representation. Notably, ordinality did *not* arise spontaneously in the apes, even though their numerals had been learned in order (Boysen et al., 1993; Tomonaga et al., 1993); they all required significant amounts of training.

Given that both apes and Alex understood symbolic representation, why was Alex the only subject to spontaneously demonstrate ordinality? As noted several times already, his use of symbols would not have qualified as language under any definition. The issue is one that I will discuss after presenting additional evidence of Alex’s numerical capacities.

#### Addition and More About Zero

Like Sheba (Boysen and Berntson, 1989), Alex also spontaneously demonstrated the ability to sum sets of objects and label that sum (Pepperberg,2006b). Initially, Alex was presented with two cups placed on a tray, under which various quantities of objects were hidden from view. He was briefly (either 2–3 or 10–15 s, depending upon the experiment) shown each quantity, after which the cup covering that quantity was replaced. After both cups were replaced, he was asked about the total number of objects, which could vary from 0 to 6. For all but a few control trials, the objects varied in mass and contour, so that Alex had to respond on the basis of number. Under the shorter time constraints, his accuracy for all sets, with the exception of 5 + 0, was just below 90%. Interestingly, under the short time constraint, he consistently labeled 5 + 0 as “six,” but was 100% accurate when given the longer time to examine the sets, suggesting that he needed the additional time actually to count the sets; when prevented from counting, he used the largest label available. Including those trials when he was given more time, his accuracy was overall 90%.

Unlike Sheba (or any other subjects), he was also asked to sum 0 + 0. Again, unlike Sheba and Ai, who had had extensive training to associate a null set with a label representing zero (Boysen and Berntson, 1989; Biro and Matsuzawa, 2001b), Alex had *spontaneously* associated “none” with a null set—he had had no formal training. Thus, asking him to label the total absence of something was a test of the extent of his *untrained* abilities. Interestingly, he mostly refused to answer, as though he realized that his standard number labels would not be correct. When forced to respond, on three of eight trials he eventually responded “one,” using the smallest quantity label he possessed. Note that Ai also sometimes confounded 0 and 1 (Biro and Matsuzawa, 2001b). Alex’s responses demonstrated that his overall understanding of the use of “none” for zero (i.e., with respect to both this and the earlier study) was comparable to that of a child just learning the concept, or of humans in cultures that do not see zero as a quantity to be labeled (Bialystok and Codd, 2000). Moreover, his occasional use of “one” clearly demonstrated that he was not simply saying “none” when he didn’t know what else to say. Unfortunately, for various reasons, we did not pursue training of his use of “none” to represent zero.

We did, however, pursue his understanding of addition. In a subsequent study, we extended questions to sets totaling to eight, to adding three rather than two hidden sets (which required additional memory), and to asking him to add hidden Arabic numerals rather than sets of objects (Pepperberg, 2012). Although his death precluded testing on all possible arrays, his accuracy was statistically significant, not subject to the vagaries of an ANS, and suggested that his capacities with respect to addition were, like those of Sheba (Boysen and Berntson, 1989), spontaneously transferrable from object sets to symbolic representations of the sets. Alex’s data, however, involved sums slightly beyond those of chimpanzees.

#### Inference of Cardinality From Ordinality

One aspect that seemed to be unique to all the non-humans during their acquisition of symbolic number labels was the lack of the so-called “bootstrapping” process that is present in young children (Carey, 2009): although, as noted earlier, the process by which children learn their first few numbers (1–4) is extremely slow (i.e., proceeds over the course of several years), they also simultaneously learn a number line—they learn to state their numerals in a specific order—even though initially the line may make little sense and the order in which they recite their numerals can be variable (Siegel, 1982; Fuson, 1988). Eventually, they learn the successor function—the ordering of their numerals stabilizes and they realize that the value of each digit in their number line is exactly one more than the previous digit—and then the bootstrapping process engages: without any further instruction they infer the meaning of numbers above 4 from this number line. In contrast, no non-human showed savings in learning as the successive numerals 5, 6, 7, etc. were added to their repertoire.

As we noted above, however, Alex’s labels were initially all trained vocally, and acquisition of each label required that he learn to produce the various sounds involved. Thus, his slow acquisition of larger numbers might have been a reflection merely of this difficulty in vocal acquisition. My colleague and I set out to test this possibility (Pepperberg and Carey, 2012).

We taught Alex to identify, vocally, Arabic numerals 7 and 8 in the absence of their respective quantities (an almost year-long process), trained him that 6 < 7 < 8 (a rapid, ∼2 month process, even when interspersed with other tasks), then tested how 7 and 8 related to his other Arabic labels (Pepperberg and Carey, 2012). If he inferred the new complete number line, he could be tested on whether he, like children (≥4 years old), *spontaneously* understood that “seven” represented exactly one more than “six,” that “eight” represented two more than “six” and one more than “seven,” by labeling appropriate physical sets on *first* trials. Data already showed he knew that the label “six” represented six items exactly, not approximately (Pepperberg and Carey, 2012); if he succeeded, we could claim that he induced cardinal meanings of “seven” and “eight” from their ordinal positions on an implicit count list, something *no* ape (although evolutionarily closer to humans) had yet achieved.

Alex learned the novel symbolic Arabic numeral labels, placed them appropriately in his inferred number line *without* training, and quantified, on first trials, novel sets of seven and eight physical items—he did *not* have to be taught the relationship between the labels and the novel sets (Pepperberg and Carey, 2012). Thus, he responded as would children, and in a way that has *not* yet been demonstrated in any other non-human.

#### Why Did Alex Differ From Other Non-humans?

According to Premack (1983), symbolic representation of number should have been adequate to enable Ai and Sheba to infer ordinality from cardinality and vice versa. According to Macphail (1987), only full human language would have allowed any of the non-humans to succeed, explaining the failure of the apes but not the success of the parrot. How do we resolve this conundrum? Could the *extent* of Alex’s symbolic representation be the issue? Might it be that Alex’s symbolic representation abilities, although far from encompassing the range of abilities that define full human language, was more ‘language-like’ than that of the other non-humans that were trained with numbers?

Sheba’s symbolic representation was limited solely to that of numerical sets. However, her representation abilities in this realm were robust—her ability to demonstrate spontaneous addition of novel combinations of Arabic numerals showed more than a simple association between a particular symbol and a particular set (Boysen, 1993). It is nevertheless possible that her limited range of symbolic understanding was not sufficient to enable the emergence of ordinality-cardinality comprehension. Furthermore, unlike children, she had never explicitly been taught a number line, and thus had no reason to expect any relationship between numbers and ordered lists.

Ai, in contrast, had had considerable training on the labeling of objects and colors (Matsuzawa, 1985), and in fact she was often required to label all three attributes of a set. However, she, like Sheba, also had not been taught an ordered list before, or simultaneously along with, training on the cardinal values of each numeral.

Alex had been taught labels for objects, materials, colors, and shapes (with respect to numbers of corners) before being taught numbers, but also was not trained with a number line. Moreover, Alex’s number labels were not even trained in order, as noted above. Alex had, however, much more overall training on symbolic representation. He was not always asked to label everything about a set (e.g., if given three blue keys, to state “three blue key” like Ai; Matsuzawa, 1985), but had to parse his sets with respect to specific categorical labels: he could be asked “How many?” and had to respond “three key” *or* “What color?” and respond “blue key” *or* “What shape?” and respond “4-corner key” (“four-corner” being Alex’s label for square items) for the same set (Pepperberg, 1983, 1999). He had also been trained on abstract concepts of same/different, such that he had to look at a pair of items and respond not as to whether they were identical or not, but, in accordance with the specific question (“What’s same?” versus “What’s different?”), provide the label of the one appropriate attribute (e.g., “color,” “shape,” “matter”; Pepperberg,1987a). Alex, therefore, had to have acquired a more complex understanding of how his labels—vocal symbols—represented the world compared to the other non-human subjects. He knew not only that “green” was associated with, for example, both a specific key and a bean, but also that it was, along with a specific subset of other labels, hierarchically grouped under another label, “color,” and likewise for his various shape, object, and material labels. His use of the order attribute + noun when multiple labels were required for identification arose through observing such use by his trainers. Alex had also already begun to parse individual labels with respect to beginnings and endings; for example, using “banerry” (banana/cherry) for an apple, and producing a label such as “carrot”—after hearing it only briefly—from his existing labels “key” and “parrot,” as well as other spontaneous rearrangements in which he carefully parsed and appropriately edited beginning and endings (e.g., “grape” to “grate” to “grain” to “chain” to “cane,” etc., Pepperberg, 1999). Later research provided additional examples (e.g., “spool” from the “s” sound and “wool”; Pepperberg, 2007). Thus, he had acquired some limited understanding of order that he might have abstracted for use with numbers. Notably, other apes that had learned something about label order (e.g., “put x in y,” Kanzi; Savage-Rumbaugh and Lewin, 1994), were not trained or tested on number concepts, nor were Herman or Schusterman’s cetaceans to any extent (see, however, Mitchell et al., 1985), and therefore comparative data are lacking.

Of course, the question still arises as to whether we can ever determine the extent to which symbolic representation—whether in the absence of ordering or with only limited understanding of ordering (rudimentary syntax)—can affect changes in cognitive processing in non-humans. A recent study suggests that to have such an effect, even symbolic representation by itself must be rich and varied, and not be a simple case of associative learning. This project involved extremely limited symbolic learning and a match-to-sample task (Bowden et al., 2019). Here researchers compared children’s (ages 3–5) and monkeys’ abilities to learn to use different icons to represent different types of matching strategies—for example, a circle meant match with respect to color whereas a cross meant match with respect to shape. Both sets of subjects learned the basic rule, but only the children could generalize to novel colors and shapes. Although children in this age range still lack full expressive language and are just beginning to learn about symbols (see discussion in Deloache, 2004), their levels of symbolic representation far outstrip those of monkeys that had been trained only on two specific symbols. Thus, not only symbolic representation, but also the extent and richness of this representation, seem critical in enabling non-humans to succeed on various tasks designed to examine their intelligence.

## A Role for Formal Syntax?

Obviously, some of what I have described so far could occur only in a subject that always had its entire repertoire available for use, that was a vocal learner, and that had human interaction for extended periods outside of training and testing sessions such that spontaneous expressions could both be noted and evoke responses from caretakers. The complicated tasks about which I have written, however, do not require formal syntax. Thus, despite all my comments on aspects of communication that could be considered to involve some type of ordering, I have not yet commented to any extent on Macphail’s basic claim with respect to the effects of the formal syntax of human language on cognitive processing. The reason for my hesitancy is the paucity of existing data and the dearth of recent (and the poor prospect for future) studies on interspecies communication, making the probability of acquiring additional knowledge unlikely (note Pepperberg, 2017).

What we currently know is of limited value: in the laboratory, some species that have learned symbolic representation have also learned something about rule-governed behavior, something clearly much simpler than formal syntax. Have researchers instilled this behavior or does it build on something already existent in nature? Some level of rule-governed behavior may exist for some species in the wild but not for others. Cetaceans learned to respond to particular orderings of symbols, but evidence for order-related meaning in their natural communication system is lacking (review in Suzuki and Zuberbühler, 2019). For some bird species that learn their songs, note and syllable order is crucial for meaning; for other species it is not (see review in Weisman and Ratcliffe, 1987). Such may also be the case for certain bird calls—in particular instances, when the order of the elements is altered, birds fail to respond in playback tests (Suzuki et al., 2019), suggesting that some sort of rules for the production and comprehension of vocalizations exist in species separated from humans by over 300 million years of evolution (Hedges et al., 1996). Evidence for what could, in some sense, be considered combinatorial order in non-human primate vocalizations in the wild has been demonstrated only in a few—primarily monkey—species (reviewed in Zuberbüler and Neumann, 2017; Suzuki and Zuberbühler, 2019), but so far seems to be used mostly in terms of modifying the level of communicative intent. Kanzi, a bonobo, demonstrated rule-governed behavior in symbolic comprehension in the laboratory (Savage-Rumbaugh and Lewin, 1994), but no evidence for such behavior has yet been discovered in the wild. Whether meanings of parrot vocalizations in the wild depend on order has not been examined, but Alex exhibited clear—if limited—sensitivity to order in various non-numerical aspects of his vocal symbolic behavior (e.g., his ability to segment, Pepperberg, 2007, his use of sentence frames “I want X” and “Wanna go Y,” where he knew that an object “X” must follow “want” but a location “Y” must follow “wanna go,” Pepperberg, 1999). Such comprehension could have been transferred to his understanding of, for example, numerical label order. Another parrot, Griffin, has also demonstrated some rule-governed behavior, as shown during a study of his acquisition of label order (Pepperberg and Shive, 2001). However, evidence for formal syntax, as opposed to simple rule-governed behavior (what can be seen as a proto-syntax) is still lacking.

These findings lead to a number of questions, particularly with respect to syntax. Does the ability to learn rule-governed behavior patterns in addition to symbolic representation simply indicate higher processing power, such that non-humans that are capable of acquiring such competence would therefore be expected to succeed on more complex tasks, and that humans with their demonstration of fully syntactic language are consequently at the apex of such behavior? Or does acquisition of rule-governed behavior in addition to symbolic representation even affect how the individual can process information? Or have brains and corresponding behavioral complexity evolved in lock-step, each synergistically supporting the next evolutionary stage (Pepperberg, 2007, 2010)? We have yet to fully determine the answers to these questions. It is likely that the effect of syntax on intelligent behavior is not easily specified, nor is the type of non-linguistic task for which formal syntax would be necessary.

## Conclusion

My overall conclusion is that differences *do* exist among various species’ abilities, that these differences are not due entirely to contextual variables, but that when individuals of these species are given appropriate training, the differences are not as great as we once may have thought. The training cannot, however, merely be with respect to simple associations between a limited number of labels and their corresponding items, but must be rich enough to encompass concepts and enable the subjects to transfer the learned concepts to novel situations. Addition of some level of rule-governed behavior would also seem important.

However, one additional issue must also be addressed when looking at the differences between humans and all other species as well as across non-human species, and when looking at the effects of language or at least symbolic representation on performance: most of the tasks that Macphail describes for comparing intelligence across species involve detecting contingencies, and little else; however, “…tasks likely to be relevant to comparative psychologists interested in intelligence” (Macphail, 1987)—tasks that actually involve more complex forms of information processing—are all designed by humans, generally are based on tasks that are appropriate for, and presented to, adult humans and/or human children, and thus are inherently biased in favor of humans who have language. Several original commentators argue the point about how the tasks proposed by Macphail fail to examine many of the more interesting qualities of information processing, but none of the commentaries fully examine the extent to which symbolic representation—or the lack thereof—may affect how well any tasks can be solved by non-humans when these are tasks that humans design from their point of view as language learners, or even how well the tasks designed for non-humans by humans will actually test what they are designed to test. From the studies I have described, it is obvious that I am not against using human-based tasks to test non-humans. My data do, however, suggest the existence of inherent biases in such tasks, given how training non-humans on symbolic representation turns out to be so important for their ability to succeed on these tasks. As researchers, we need to be aware of such issues when making claims about non-human intelligence.

In sum, despite the still-contemporary feel of “The Comparative Psychology of Intelligence,” article and commentaries, much has indeed changed in the intervening 30 years. In 1987, an extremely high number of papers in psychological journals (see Burghardt, 2006) still generally involved only rodents or pigeons performing some kind of experiment using operant conditioning. Now, we can access studies about everything from ants to lizards to horses to elephants that examine everything from visual illusions to social cooperation to spatial orientation to delayed gratification. New techniques have given us access to levels of neurophysiological and neuroanatomical information that enable incredibly detailed cross-species analyses. We have new statistical tools and modeling algorithms—and the computational power to use them—that few could have foreseen. Many of us studying non-humans are asked to collaborate on projects spanning AI to SETI. Macphail (1987) may not have foreseen the actual future of comparative psychology, but we must give him credit for instigating a variety of controversies, stimulating the wide-ranging discussions, and generating the types of challenges that have led to many new avenues of research.

## Author Contributions

The author confirms being the sole contributor of this work and has approved it for publication.

## Conflict of Interest

The author declares that the research was conducted in the absence of any commercial or financial relationships that could be construed as a potential conflict of interest.
